# Effects of differentially expressed microRNAs induced by rootstocks and silicon on improving chilling tolerance of cucumber seedlings (*Cucumis sativus* L.)

**DOI:** 10.1186/s12864-023-09337-x

**Published:** 2023-05-10

**Authors:** Qiang Ma, Chenxu Niu, Chao Wang, Chunhua Chen, Yan Li, Min Wei

**Affiliations:** 1 College of Horticultural Science and Engineering, Shandong Agricultural University, Taian, 271018 China; 2 Scientific Observing and Experimental Station of Environment Controlled Agricultural Engineering in Huang-Huai-Hai Region, Ministry of Agriculture and Rural Affairs, Taian, 271018 China; 3 State Key Laboratory of Crop Biology, Taian, 271018 China; 4 Collaborative Innovation Center of Fruit & Vegetable Quality and Efficient Production in Shandong, Taian, 271018 China

**Keywords:** MicroRNA, Chilling tolerance, Rootstock, Silicon, Cucumber

## Abstract

**Background:**

Rootstocks can improve the chilling tolerance of grafted cucumbers, but their effectiveness varies. Rootstocks with strong de-blooming capacity may result in lower chilling tolerance of grafted cucumbers compared to those with weak de-blooming capacity, while also reducing the silicon absorption. However, it remains unclear whether this reduction in chilling tolerance is due to differences in rootstock genotypes or the reduction in silicon absorption.

**Results:**

The chilling tolerance of cucumber seedlings was improved by using rootstocks and silicon nutrition. Rootstocks had a more significant effect than silicon nutrition, and the weak de-blooming rootstock ‘Yunnan figleaf gourd’ was superior to the strong de-blooming rootstock ‘Huangchenggen No. 2’. Compared to self-rooted cucumber, twelve miRNAs were regulated by two rootstocks, including seven identical miRNAs (novel-mir23, novel-mir26, novel-mir30, novel-mir37, novel-mir46, miR395a and miR398a-3p) and five different miRNAs (novel-mir32, novel-mir38, novel-mir65, novel-mir78 and miR397a). Notably, four of these miRNAs (novel-mir38, novel-mir65, novel-mir78 and miR397a) were only identified in ‘Yunnan figleaf gourd’-grafted cucumbers. Furthermore, six miRNAs (miR168a-5p, miR390a-5p, novel-mir26, novel-mir55, novel-mir67 and novel-mir70) were found to be responsive to exogenous silicon. Target gene prediction for 20 miRNAs resulted in 520 genes. Functional analysis of these target genes showed that ‘Yunnan figleaf gourd’ improves the chilling tolerance of cucumber by regulating laccase synthesis and sulfate metabolism, while ‘Huangchenggen No. 2’ and exogenous silicon reduced chilling stress damage to cucumber by regulating ROS scavenging and protein protection, respectively.

**Conclusion:**

Among the identified miRNAs, novel-mir46 and miR398a-3p were found in cucumbers in response to chilling stress and two types of rootstocks. However, no identical miRNAs were identified in response to chilling stress and silicon. In addition, the differential expression of novel-mir38, novel-mir65, novel-mir78 and miR397a may be one of the important reasons for the differences in chilling tolerance of grafted cucumbers caused by two types of rootstocks.

**Supplementary Information:**

The online version contains supplementary material available at 10.1186/s12864-023-09337-x.

## Background

Cucumber (*Cucumis sativus* L.) is an important vegetable crop grown in protected cultivation facilities. It is native to the tropics and is considered a cold-sensitive crop [1]. In some northern regions, low winter and spring temperatures can seriously affect the normal growth of cucumbers in protected cultivation facilities, leading to a reduction in both yield and quality.

Grafting is a significant agronomic technique that can improve the yield and stress tolerance of cucumber plants [2]. In the past, *Cucurbita ficifolia* Bouché has been the preferred rootstock due to its superior stress tolerance. However, the use of *Cucurbita ficifolia* Bouché as a rootstock leads to a thick layer of bloom on the surface of grafted cucumber fruit, which ultimately reduces the quality of the cucumber [3]. In recent years, *Cucurbita moschata* Duch has been gradually increased to improve the quality of cucumbers [4]. However, cucumbers grafted with *Cucurbita moschata* Duch have been found to have lower chilling tolerance compared to those grafted with *Cucurbita ficifolia* Bouché [5, 6].

Several studies have shown that the amount of bloom on the fruit surface of cucumber is related to the ability of plants to absorb and metabolize silicon [7, 8]. Silicon is a beneficial element for plant development, as it increases cucumber yield and mitigates the negative impacts of abiotic stressors [9–11]. It has been speculated that the chilling tolerance of grafted cucumber may be related to its absorption and metabolism of silicon. However, it is unclear how rootstock genotypes and silicon nutrition differ in their respective roles and molecular mechanisms in improving the chilling tolerance of cucumber.

Plant microRNAs (miRNAs) are endogenous non-coding small RNAs of 21–24 nucleotides (nt) in length that are highly conserved among species and play an essential role in gene regulation [12]. miRNAs can target single or multiple genes to regulate plant responses to stressful environments [13]. Studies have shown that low temperatures can alter miRNAs expression in various plants, such as wheat [14], Arabidopsis [15], and tomato [16]. In addition, some studies have suggested that regulating miRNAs expressions through rootstocks and exogenous can improve plant stress tolerance [17–20]. These results have shown that miRNAs play an essential role in the response of plants to chilling stress.

The chilling tolerance of cucumber is influenced by rootstocks and silicon nutrition. However, determining whether the main cause of variation in the chilling tolerance of grafted cucumber is due to the rootstock genotype or silicon nutrition requires more systematic and in-depth studies. In this study, we used ‘Xintaimici’ cucumber together with two different rootstocks: the strong de-blooming rootstock ‘Huangchenggen No. 2’ (*Cucurbita moschata* D.) and the weak de-blooming rootstock ‘Yunnan figleaf gourd’ (*Cucurbita ficifolia* B.) as experimental materials. We employed high-throughput sequencing technology to identify miRNAs regulated by rootstocks and silicon under chilling stress. Our objective was to investigate the cause of altered chilling tolerance in cucumber from the perspective of miRNAs.

## Results

### Rootstock types and silicon nutrition on chilling tolerance of cucumber seedlings

The growth index and chilling injury index of cucumber seedlings in five treatments were measured to visually demonstrate the difference between the two rootstocks and silicon nutrition in reducing chilling injury (Table 1). In the TC/CC comparison pair, chilling stress inhibited the normal growth of cucumbers and caused severe chilling injury to the leaves. In the TS/TC, TH/TC and TY/TC comparison pairs, the application of two rootstocks and silicon nutrition reduced chilling injury, with rootstocks being more effective than silicon nutrition. Notably, the ‘Yunnan figleaf gourd’ showed the most significant reduction in chilling injury among the rootstocks.

**Table 1:** Effects of rootstocks and silicon on chilling tolerance of cucumber seedlings

Treatments	Plant height (cm·plant^− 1^)	Stem diameter (mm·plant^− 1^)	Leaf area (cm^2^ ·plant^− 1^)	Shoot Fresh weight (g·plant^− 1^)	Shoot Dry weight (g·plant^− 1^)	Chilling damage index (score)
CC	5.43 ± 0.31a	5.94 ± 0.17b	102.43 ± 5.19a	8.90 ± 0.64ab	1.19 ± 0.06a	0.00 ± 0.00d
TC	2.84 ± 0.14d	4.83 ± 0.14c	64.05 ± 1.02c	5.88 ± 0.17c	0.90 ± 0.03b	0.53 ± 0.11a
TS	3.45 ± 0.03c	5.25 ± 0.06c	71.32 ± 1.70c	6.26 ± 0.11c	0.93 ± 0.02b	0.35 ± 0.07b
TH	4.35 ± 0.08b	5.97 ± 0.12b	90.36 ± 3.32b	7.85 ± 0.15b	1.14 ± 0.03a	0.28 ± 0.05bc
TY	5.31 ± 0.10a	6.56 ± 0.11a	107.69 ± 3.62a	9.38 ± 0.14a	1.26 ± 0.03a	0.21 ± 0.03c

**CC**: Self-rooted cucumber seedlings were grown in nutrient solution and maintained at 28 ± 1 °C/18 ± 1 °C. **TC**: Self-rooted cucumber seedlings were grown in nutrient solution and maintained at 10 ± 1 °C/5 ± 1 °C. **TS**: Self-rooted cucumber seedlings were grown in nutrient solution with 0.5 mmol·L^− 1^ Na_2_SiO_3_·9H_2_O and maintained at 10 ± 1 °C/5 ± 1 °C. **TH**: ‘Huangchenggen No. 2’-grafted cucumber seedlings were grown in nutrient solution and maintained at 10 ± 1 °C/5 ± 1 °C. **TY**: ‘Yunnan figleaf gourd’-grafted cucumber seedlings were grown in nutrient solution and maintained at 10 ± 1 °C/5 ± 1 °C. Different letters indicate significant differences at 0.05 level. The same is as follows

### Small RNA regulated by rootstock types and silicon nutrition under chilling stress

**Fig. 1:**
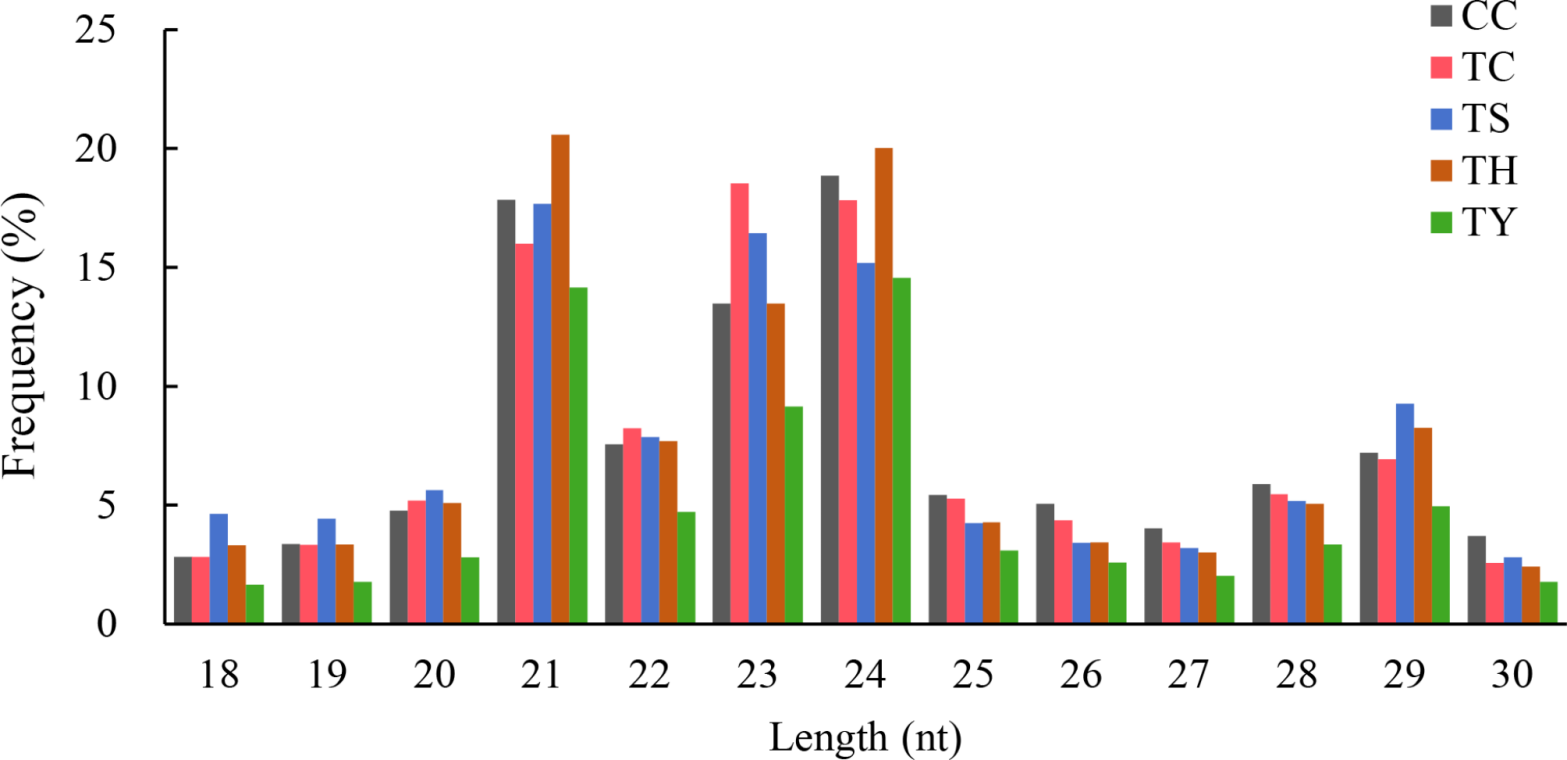
Length distribution of sRNA in CC, TC, TS, TH and TY libraries. Y-axis represents percentages of sRNAs identified in the study; X-axis represents the length of sRNAs. Five libraries are shown in different colours

We constructed five RNA libraries (CC, TC, TS, TH and TY) to investigate the differential expression of small RNAs (sRNAs) in cucumber seedlings under chilling stress in response to rootstocks and silicon. The raw reads identified in these libraries were 13,472,603, 12,525,103, 12,975,897, 14,870,839, and 14,685,213, respectively (Table 2). After removing contaminating and low-quality sequences, 13,258,990 (98.41%), 12,315,436 (98.33%), 12,722,672 (98.05%), 14,607,618 (98.23%) and 14,377,447 (97.90%) clean reads were retained, respectively, and their lengths were calculated (Fig. 1). The results showed that the lengths of sRNAs in five libraries were mainly concentrated in 21 nt and 24 nt, accounting for 17.25% and 17.30% of the total reads, respectively, followed by sRNAs with the length of 23 nt, accounting for 14.22%.

**Table 2:** Summary of cleaning data in CC, TC, TS, TH and TY libraries

Type	CC	TC	TS	TH	TY
Total reads	13,472,603	12,525,103	12,975,897	14,870,839	14,685,213
N% > 10%	234	160	159	450	354
Low quality	35,070	27,926	23,355	46,677	48,232
3`adapter null	146,044	139,606	198,688	164,046	204,200
5`adapter contaminants	11,458	12,153	14,893	21,265	19,568
Poly (A/T/G/C)	20,807	29,821	16,130	30,784	35,413
Clean reads	13,258,990	12,315,436	12,722,672	14,607,618	14,377,447

Note: ‘N’ indicates reads for which base information could not be determined

All clean reads from the five libraries were then aligned with genome databases to classify the remaining sRNAs (Table 3). The results showed that 9,547,918 (53.4%), 8,366,100 (64.1%), 10,000,041 (67.62%), 10,628,208 (72.76%) and 9,993,536 (69.51%) of the reads in five libraries mapped to the cucumber genome database, respectively. Among them, 5,047,596, 4,339,259, 5,855,676, 5,093,037, and 4,231,038 reads in five libraries were annotated as tRNA, rRNA and other non-coding RNAs, respectively. In addition, 277,069, 232,908, 239,674, 333,648 and 321,294 clean reads matched exons or repetitive regions in the five libraries, respectively. Finally, among the remaining clean reads in each library, 548,451, 452,785, 762,301, 735,048 and 679,968 reads could be matched to known miRNAs in miRBase. The remaining 3,275,890, 2,839,006, 2,712,306, 3,915,178 and 4,217,851 reads in each library reached the non-coding region of the cucumber genome and might contain novel miRNAs.

**Table 3:** Categorization of small RNAs in CC, TC, TS, TH and TY libraries

Category	CC	TC	TS	TH	TY
Clean reads	13,258,990	12,315,436	12,722,672	14,607,618	14,377,447
Map to cucumber genome	9,547,918	8,366,100	10,000,041	10,628,208	9,993,536
Rfam^*a*^	5,047,596	4,339,259	5,855,676	5,093,037	4,231,038
Repeat	116,550	95,787	96,759	145,595	149,364
Exon	160,519	137,121	142,915	188,053	171,930
miRBase	548,451	452,785	762,301	735,048	679,968
Unannotated	3,275,890	2,839,006	2,712,306	3,915,178	4,217,851

Rfam^*a*^, reads mapped to plant rRNA, tRNA and snoRNA in Rfam

### Differentially expressed miRNAs regulated by rootstocks and silicon under chilling stress

We identified 64 known miRNAs belonging to 26 miRNA families in five sRNA libraries (Additional file 1: Table S1). After removing low-expressed sequences with less than 10 reads in each library for subsequent data analysis, 44 known miRNAs from 22 miRNA families were retained (Additional file 2: Table S2). Among these miRNA families, miR156/157, miR159, miR165/miR166, miR168, miR171, miR319, miR390, miR396 and miR398 had more than two members. The miR171 family had the highest number of members with five. In contrast, miRNA families such as miR160, miR162, and miR172 had only one member each. In addition, the expression of miRNA could be inferred from their reads. For example, miR159a and miR166a-3p had more than 100,000 reads in each library, indicating that they were highly expressed. However, the reads of miR390b-3p, miR858a, miR858b and miR2111a-5p were all less than 30 in all five libraries, indicating that their expression levels were significantly low. Finally, the classification of known miRNAs according to sequence length revealed that 21 nt was the major length of known miRNAs, accounting for 79%.

To identify potential novel miRNAs, we aligned the remaining reading fragments from the five libraries to the cucumber genome by using miREvo and mirdeep2 identifying 63 novel miRNAs after removing those with less than 10 reads in each library (Additional file 3: Table S3). Analysis of the length analysis of these novel miRNA revealed that 21 nt and 24 nt were the major lengths across the five libraries, accounting for 36.5% and 44.4%, respectively.

**Fig. 2:**
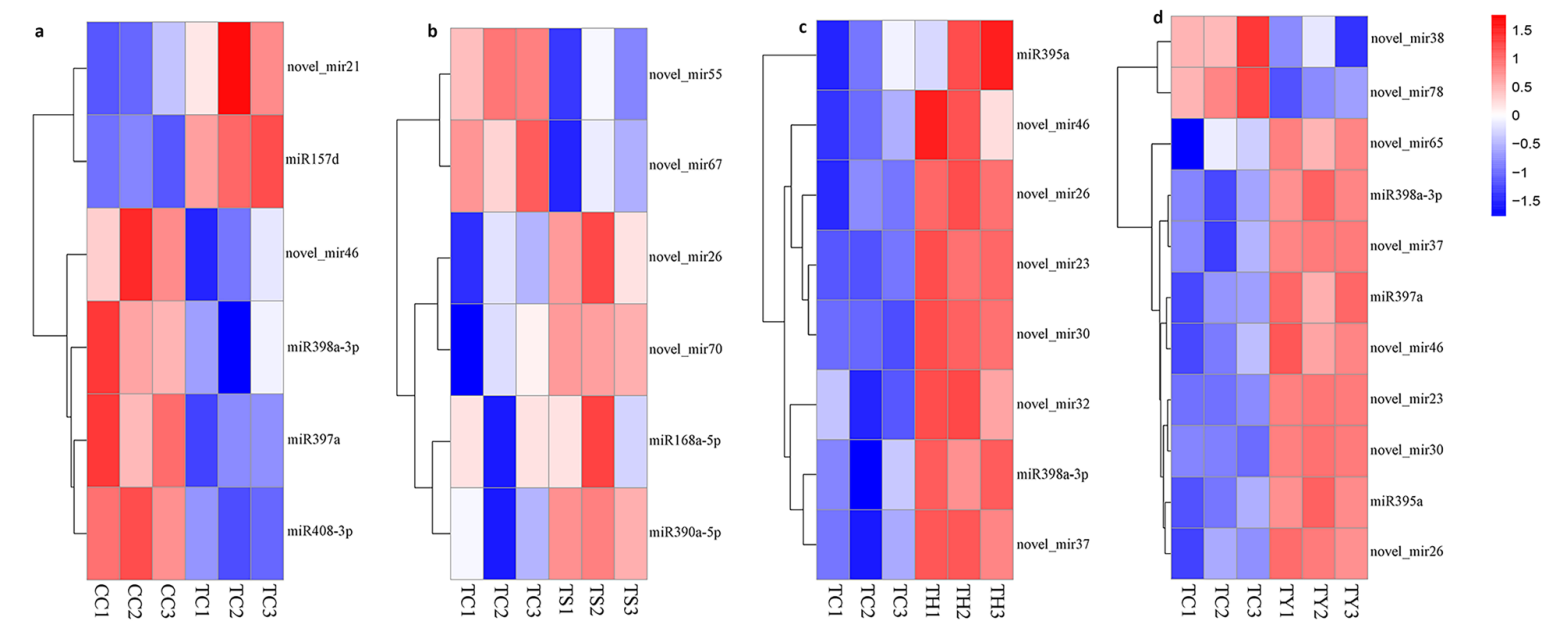
Heat map of differentially expressed miRNAs in four comparison pairs. **a** Differentially expressed miRNAs in TC/CC comparison pair. **b** Differentially expressed miRNAs in TS/TC comparison pair. **c** Differentially expressed miRNAs in TH/TC comparison pair. **d** Differentially expressed miRNAs in TY/TC comparison pair. Data are presented as heatmaps of Log2-transformed fold change. Red indicates high expression, white indicates intermediate expression, and blue indicates low expression

To investigate the effect of rootstock type and silicon nutrition under chilling stress on miRNA regulation, five libraries were divided into four comparison pairs (TC/CC, TS/TC, TH/TC, and TY/TC). The expression of miRNAs in each comparison pair was compared to obtain four groups of differentially expressed miRNAs (Fig. 2). In the TC/CC comparison pair, 6 miRNAs were identified in response to chilling stress, including 4 known miRNAs (miR408-3p, miR398a-3p, miR397a and miR157d) and 2 novel miRNAs (novel-mir21 and novel-mir46) (Fig. 2A). The TH/TC and TY/TC comparison pairs revealed 8 and 11 differentially expressed miRNAs, respectively (Fig. 2C and D). Among the miRNAs regulated by ‘Huangchenggen No. 2’, including 2 known miRNAs (miR395a and miR398a-3p) and 6 novel miRNAs (novel-mir23, novel-mir26, novel-mir30, novel-mir32, novel-mir37 and novel-mir46). There were 11 miRNAs regulated by ‘Yunnan figleaf gourd’, including 3 known miRNAs (miR395a, miR397a and miR398a-3p) and 8 novel miRNAs (novel-mir23, novel-mir26, novel-mir30, novel-mir38, novel-mir37, novel-mir46, novel-mir65, and novel-mir78). Finally, 6 miRNAs were identified from the TS/TC comparison pair, induced by exogenous silicon under chilling stress, including 2 known miRNAs (miR168a-5p and miR390a-5p) and 4 novel miRNAs (novel-mir26, novel-mir55, novel-mir67 and novel-mir70) (Fig. 2B).

**Fig. 3:**
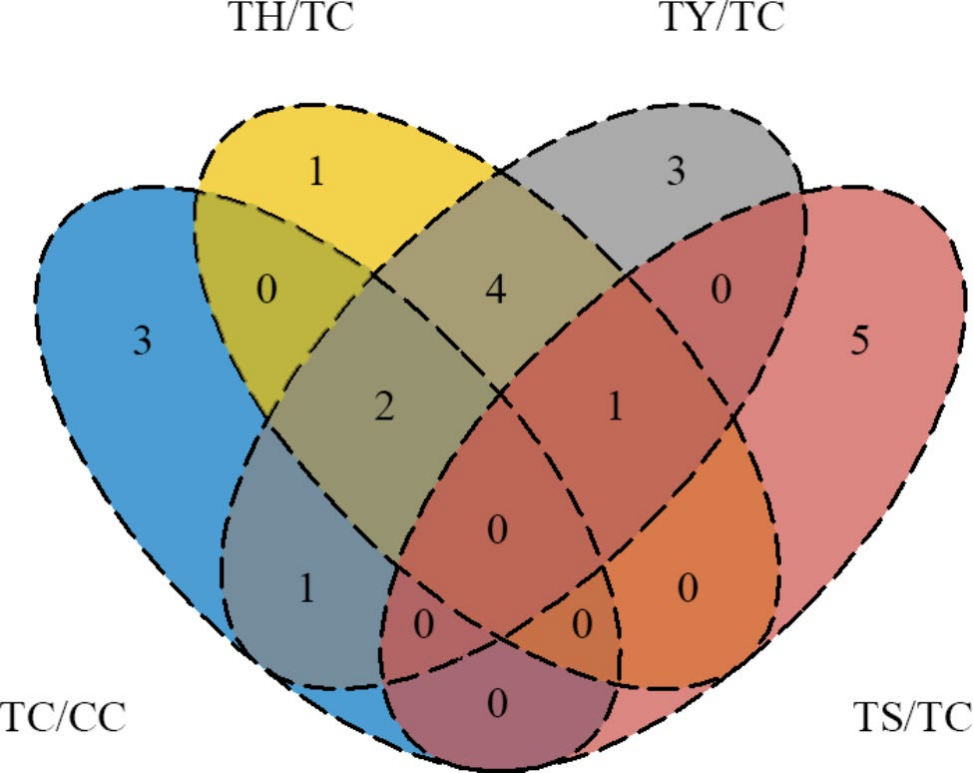
Venn diagrams for analysis of miRNAs differentially expressed in TC/CC, TS/TC, TH/TC and TY/TC comparison pairs from cucumber seedlings

The Venn diagram displayed the number of identical and differential miRNAs in four comparison pairs (Fig. 3). The results showed that 7 identical miRNAs (novel-mir23, novel-mir26, novel-mir30, novel-mir37, novel-mir46, miR395a and miR398a-3p) and 5 different miRNAs (novel-mir32, novel-mir38, novel-mir65, novel-mir78 and miR397a) were regulated by two rootstocks. Among the different miRNAs, novel-mir32 was solely identified in ‘Huangchenggen No. 2’-grafted cucumber, while novel-mir38, novel-mir65, novel-mir78 and miR397a were solely identified in ‘Yunnan figleaf gourd’-grafted cucumber. The expression of miR397a was regulated by both ‘Yunnan figleaf gourd’ and chilling stress, but the expression patterns were different. Six miRNAs (novel-mir26, novel-mir55, novel-mir67, novel-mir70, miR168a-5p and miR390a-5p) were regulated by silicon in the TS/TC comparison pair. Among them, novel-mir26 showed the same expression pattern in TY/TC and TH/TC comparison pairs. No miRNAs were identified as responsive to both low temperature and silicon in TC/CC and TS/TC comparison pairs.

### Target genes prediction and functional analysis of differentially expressed miRNAs

In our study, we identified 20 differentially expressed miRNAs in four comparison pairs, including 7 known miRNAs and 13 novel miRNAs. These miRNAs targeted a total of 520 genes, with the number of target genes per miRNA ranging from 0 to 120. Notably, miR157d had the highest number of target genes among all miRNAs (Additional file 4: Table S4).

**Fig. 4:**
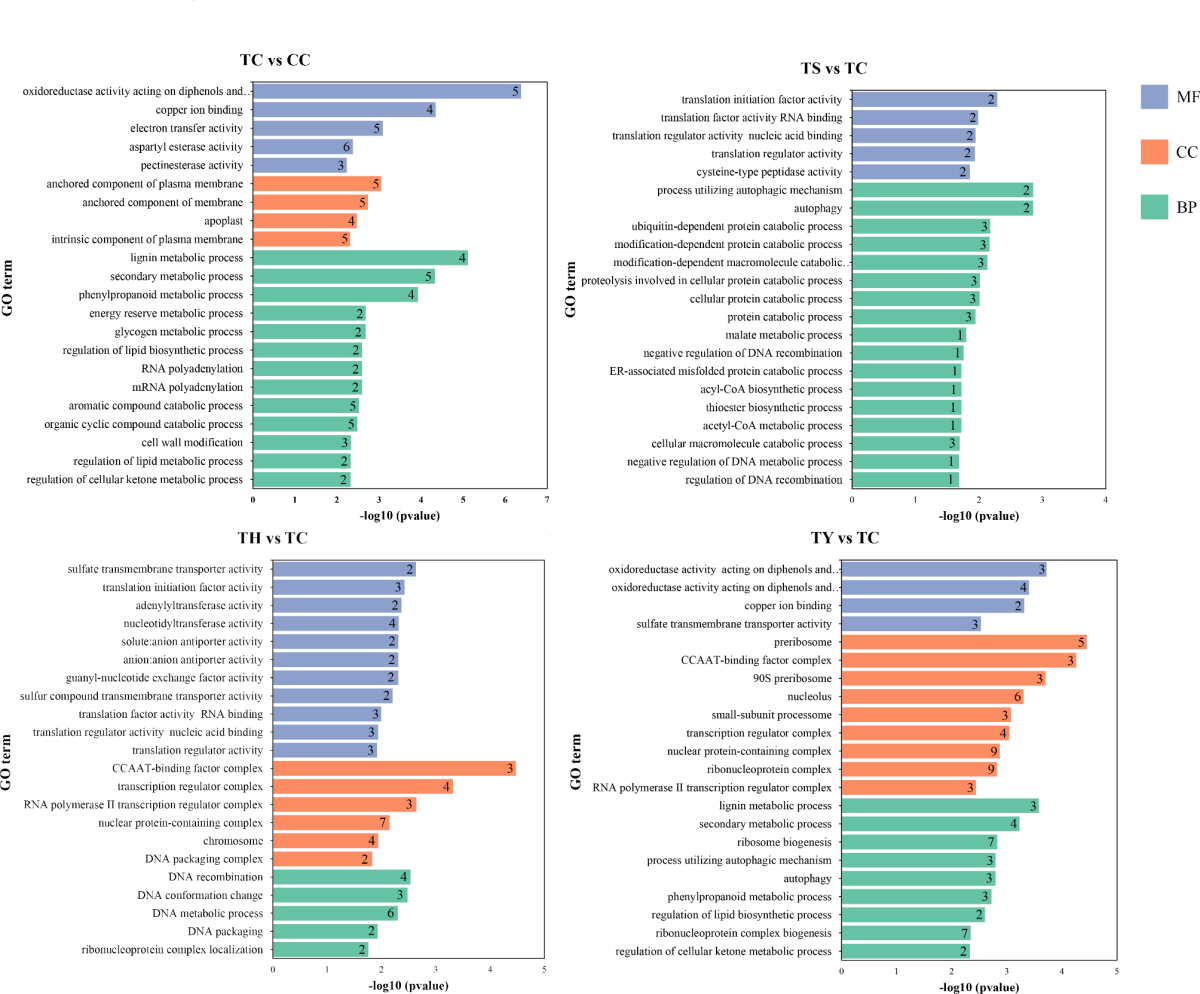
Gene Ontology annotation analysis of genes targeted by miRNAs in four comparison pairs. The vertical axis represents enriched GO terms, the horizontal axis represents the enriched *p*-value of each term, and the numbers in bars indicate the number of genes enriched in each terms. Different colours are used to distinguish cellular components, biological processes and molecular functions

These genes were subjected to GO (Gene Ontology) annotation classification analysis. In TC/CC, TS/TC, TH/TC and TY/TC comparison pairs, 140, 82, 219 and 257 target genes were assigned to GO terms, respectively. (Fig. 4). Notably, the ‘CCAAT-binding factor complex’ in Cell Component and ‘sulfate transporter activity’ in Molecular Function were commonly enriched in two grafted cucumbers. Some unique GO terms were identified in ‘Yunnan figleaf gourd’ compared to ‘Huangchenggen No. 2’, such as ‘preribosome’, ‘nucleolus’, ‘nuclear protein-containing complex’ and ‘ribonucleoprotein complex’ in Cell Component, and ‘ribosome biogenesis’ and ‘ribonucleoprotein complex biogenesis’ in Biological Process. The functional enrichment process of miRNAs regulated by silicon nutrition was significantly different from other treatments. In TS/TC comparison pair, the GO terms were mainly concentrated in ‘translation initiation factor activity’ in Molecular Function and ‘autophagy functions’ in Biological Process were significantly enriched.

**Fig. 5:**
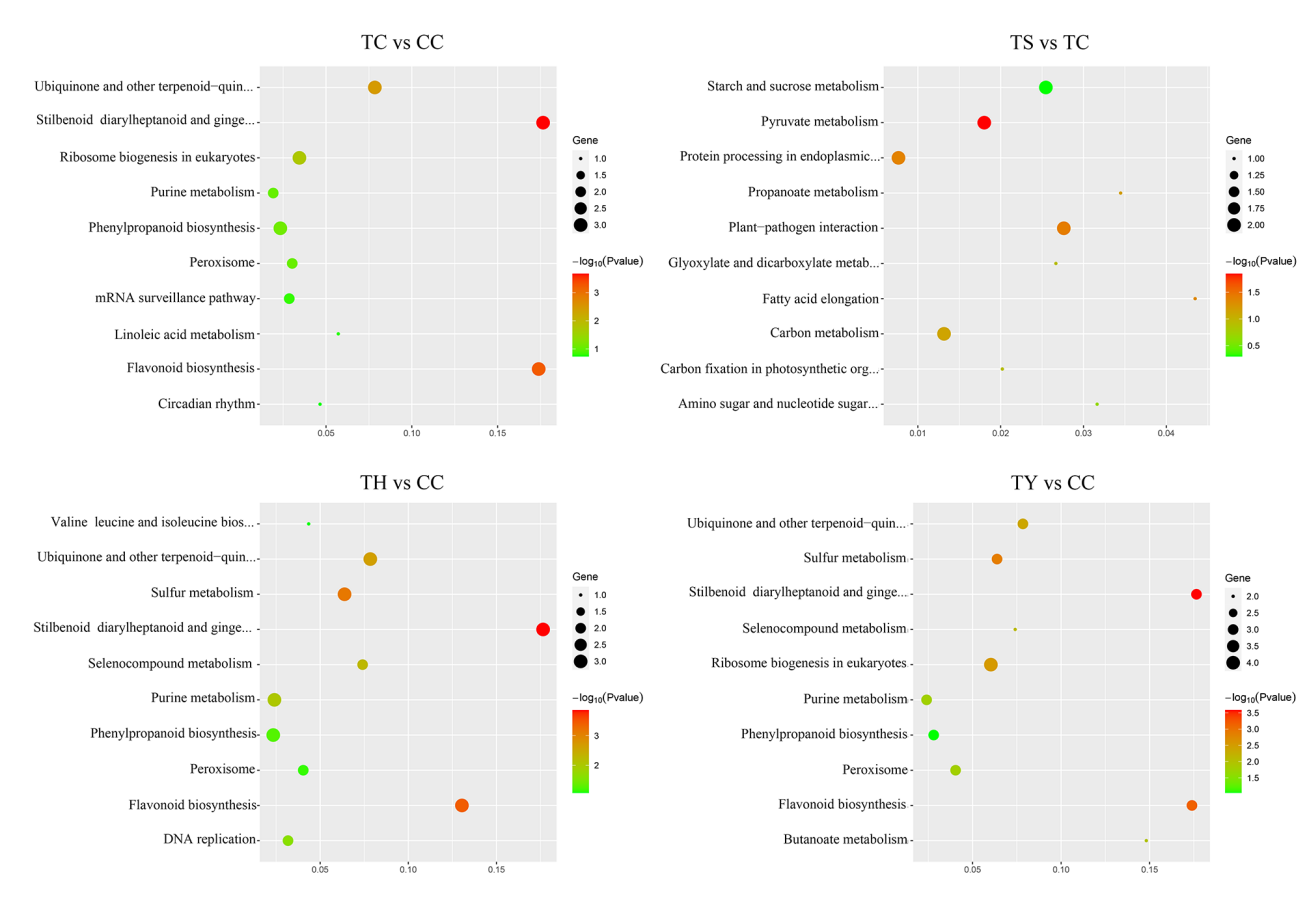
KEGG pathways of miRNAs target genes were significantly enriched in four comparison groups. The vertical axis represents the gene enrichment pathway, and the horizontal axis represents the rich factor. The size of the dot indicates the number of candidate target genes in the pathway, and the colour of the dot corresponds to different *p*-value ranges

The target genes were then subjected to KEGG (Kyoto Encyclopedia of Genes and Genome) metabolic pathway analysis. The number of enriched pathways varied among the pairs, with the highest number being 36 in TY/TC comparison pair (Fig. 5). The pathways of TH/TC and TY/TC were found to be similar, such as ‘Stilbenoid, diarylheptanoid and gingerol biosynthesis’ and ‘Flavonoid biosynthesis’ were the common pathways in both types of grafted cucumber. In addition, ‘Flavonoid biosynthesis’ was identified as a common pathway in TC/CC, TH/TC and TY/TC pairs, indicating its potential fundamental role in the response to chilling stress in different types of grafted cucumber.

### qRT-PCR verification of differentially expressed miRNAs and their target genes

**Fig. 6:**
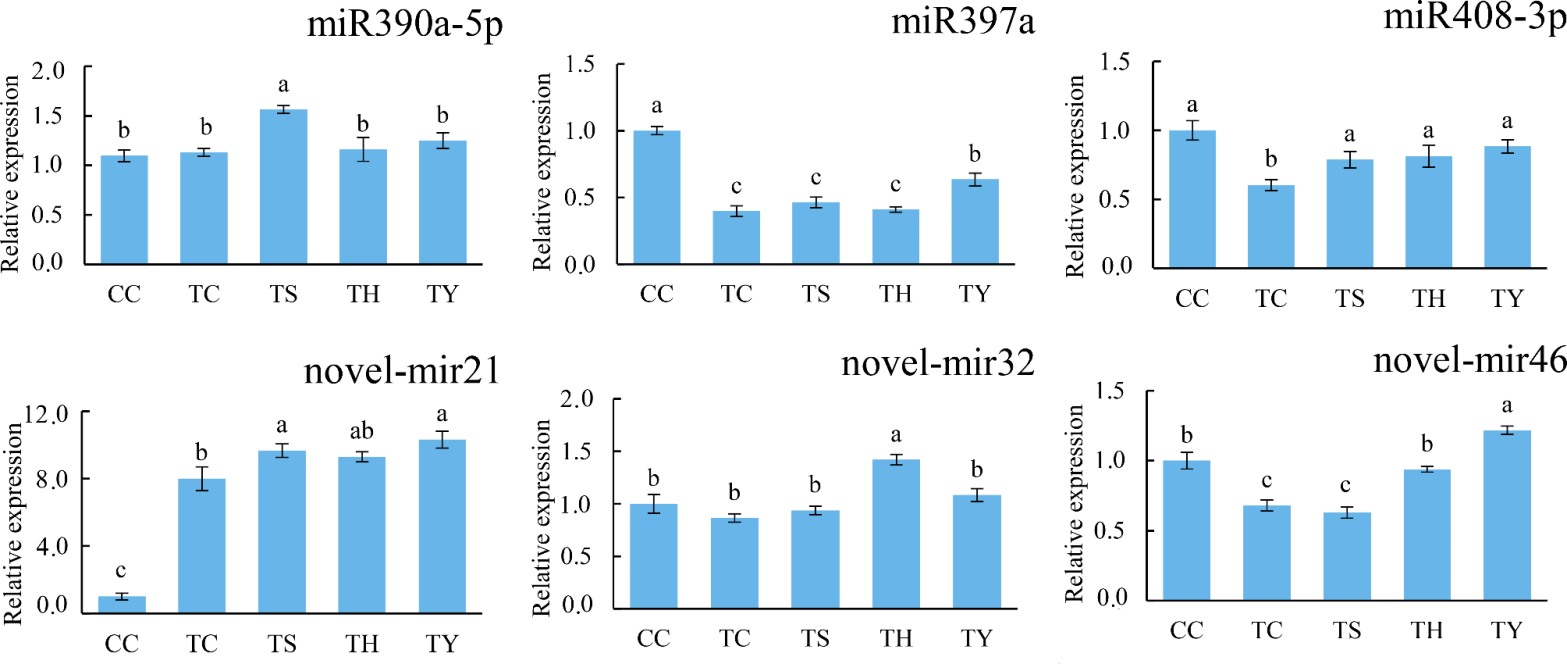
qRT-PCR expression profiling of six randomly selected miRNAs.The U6 snRNA was used as an internal control. Error bars represented the ± SD calculated from three replicates. Significant differences by Student`s t-test were indicated by different letters (*p-*value ≤ 0.05, n = 3)

**Fig. 7:**
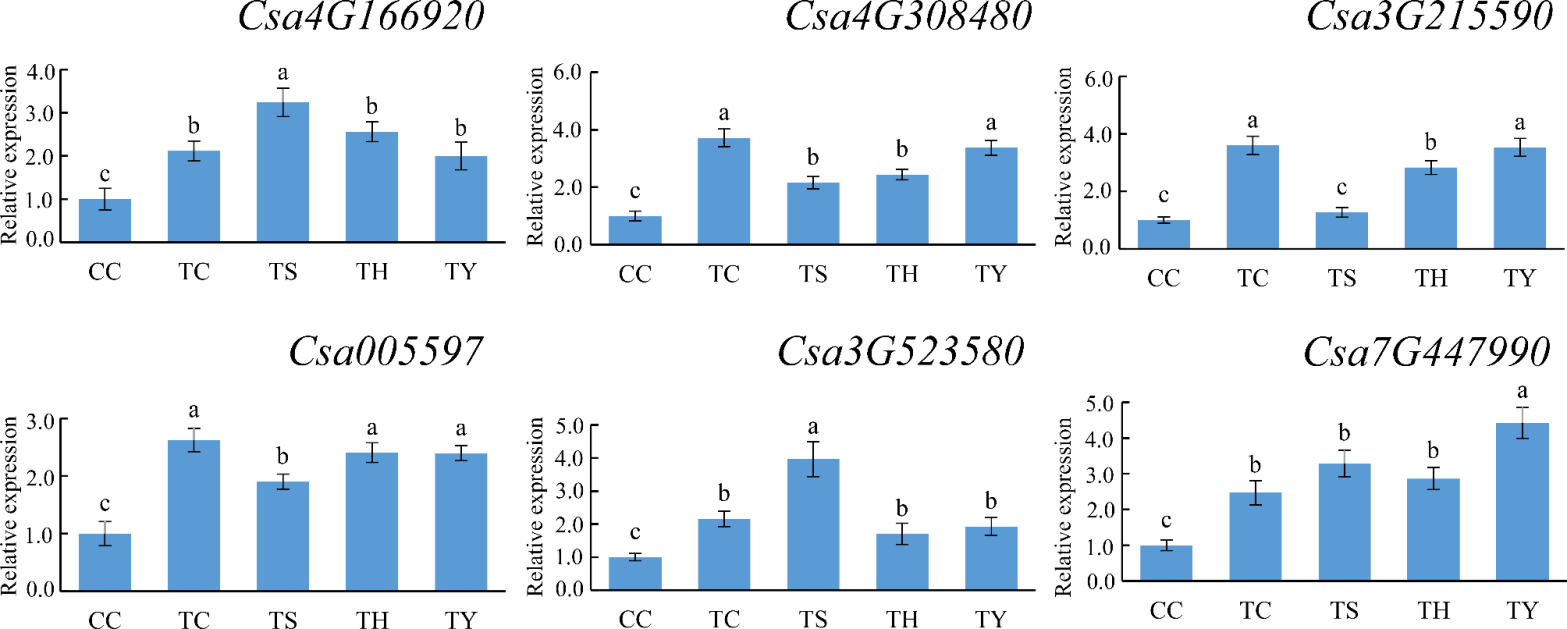
qRT-PCR expression profiling of six randomly selected target genes. The actin gene was used as an internal control. Error bars represented the ± SD calculated from three replicates. Significant differences by Student`s t-test were indicated by different letters (*p*-value ≤ 0.05, n = 3)

Three known miRNAs and three novel miRNAs were selected randomly for quantitative verification by qRT-PCR (Fig. 6). The results showed that the expression trends of the miRNAs detected by qRT-PCR were consistent with the sequencing results. For example, the expression of miR397a was down-regulated at low temperatures. Under chilling stress, ‘Yunnan figleaf gourd’ up-regulated its expression, while ‘Huangchenggen No. 2’ and silicon nutrition had no significant effect. The expression of novel-mir46 was inhibited by chilling stress and two types of rootstocks, but exogenous silicon up-regulated its expression. In addition, we quantified six target genes of differentially expressed miRNAs by qRT-PCR to explore the regulatory relationship between miRNAs and target genes (Fig. 7). The results showed that miRNAs regulated the expression of target genes through negative regulation. For example, low temperature down-regulated miR397a and up-regulated the expression of its target genes. The expression patterns of miRNAs identified by qRT-PCR and high-throughput sequencing were consistent, indicating the reliability of the sequencing results.

## Discussion

It is well known that plants can regulate the expression of genes related to chilling tolerance by mediating miRNAs in response to chilling injury [14–16]. The effects of rootstocks with different de-blooming capacities and exogenous silicon on the chilling tolerance of cucumber seedlings were significantly different (Table 1). In this study, we attempted to identify miRNAs that are regulated by two rootstocks and silicon nutrition under low temperatures, and to analyze their roles in regulating the chilling tolerance of cucumber seedlings.

### miRNAs in response to chilling stress in cucumber seedlings

The study identified 6 miRNAs (miR157d, miR397a, miR398a-3p, miR408-3p, novel-mir21 and novel-mir46) that respond to chilling stress in cucumber seedlings. Some studies have shown that miR157d is a crucial miRNA related to cotton development and up-regulated miR157 can inhibit tissue cell proliferation and cell expansion [19]. In this study, chilling stress up-regulated miR157d, which was predicted to target various genes, including *SQUAMOSA*-promoter binding protein gene family (*SP13B*, *SPL6*, *SPB1*, *SPL7* and *SPL17)*, *leucine-rich repeat receptor-like serine/threonine-protein kinase BAM1*, *cationic amino acid transporter 8* (*CAT8)*, *short-chain dehydrogenase TIC32* and *serine/arginine-rich splicing factor RSZ2*. *BAM1* and *SPL* are essential transcription factors associated with floral organ development [20, 21]. CAT8 plays an essential role in maintaining intracellular amino acid homeostasis by regulating the amino acid content inside and outside the vacuole [22]. *RSZ22* is homologous gene of human *SRSF7* factor and may be involved in pre-mRNA splicing [23]. TIC32 is an integral part of chloroplast biogenesis [24]. These results indicated that cucumber seedlings might alter the expression of miR157d to control the growth of leaf cells in response to chilling stress.

miR397 can regulate the expression of laccase, POD and L-ascorbate oxidase, or directly target *inducer of CBF expression 1* (*ICE1)* to regulate *CBF* genes, thereby participating in plant response to chilling stress [14, 25]. However, the expression patterns of miR397 in response to chilling stress vary among species. For example, the expression of miR397 decreased in wheat but increased in Arabidopsis under chilling stress [14, 15]. In this study, we observed that chilling stress suppressed the expression of miR397a in cucumber seedlings. *LAC4*, *LAC11*, *LAC17* and *aborted microspores* (*AMS)* were predicted to be the primary target genes of miR397a. Some studies have shown that inhibiting the expression of *LAC4* and *LAC17* can significantly reduce the lignin content, resulting in a significant decrease in plant tolerance to chilling stress [26].

miR398 is a highly conserved miRNA that plays a crucial role in maintaining the balance of intracellular ROS content and mitigating stress damage in plants by regulating the expression of c*opper/zinc superoxide dismutase* (*CSD1* and *CSD2*) in plants [27, 28]. In this study, miR398a-3p was inhibited by chilling stress and was predicted to target *CSD*. Some studies have shown that CSD can enhance the ROS scavenging system and promote the plant response to chilling stress [29].

miR408 can affect plant tolerance to chilling stress by regulating ROS content, lignin synthesis and intracellular copper transport [30, 31]. Its expression pattern varies depending on the type of stress, such as salt stress, chilling stress and oxidative stress inducing its up-regulation, whereas drought and osmotic stress inhibit its expression [30]. In this study, the expression of miR408 was decreased after chilling stress, and miR408 was predicted to target *Basic blue protein* (*BABL)* and *Copper-transporting ATPase PAA2*. BABL, a structural protein of anthocyanins, positively affects plant tolerance to chilling stress [32]. Similarly, PAA2 plays an essential role in maintaining normal chloroplast function by transporting copper ions into the chloroplast [33].

In addition, novel-mir21 and novel-mir46 were predicted to respond to low temperatures. Novel-mir21 was predicted to target *TCP2*, *TCP4*, *MYB33* and *A20/AN1 zinc finger domain-containing stress-associated protein 1* (*SAP1)*. The expression of *SPA1* is induced by chilling stress, and the overexpression of *SAP1* can improve the tolerance of tobacco to chilling stress [34]. TCP transcription factors can affect leaf development by regulating the cell cycle, and down-regulation of *TCP2* expression leads to leaf deformation [35]. The novel-mir46 was predicted to target *pectinesterase 3* (*PME3)*. PME3 is capable of catalyzing the demethylation of pectin, resulting in the release of free carboxyl groups, methyl groups, and protons, which can improve cell wall stiffness and plant tolerance to chilling stress [36].

### miRNAs associated with chilling tolerance affected by two rootstocks

Grafting is an important agronomic technique for improving the chilling tolerance of cucumbers. Compared with self-rooted cucumber, differential analysis of miRNAs in ‘Huangchenggen No. 2’-grafted cucumber and ‘Yunnan figleaf gorund’-grafted cucumber identified 8 and 11 different miRNAs, respectively (Fig. 2A). miR395a and miR398a-3p were identical miRNAs found in two types of grafted cucumber.

Previous studies have shown that miR395 plays a crucial role in the response of plants to salt and drought stress by regulating sulfate metabolism [37]. Recent studies have shown that miR395 expression can be altered in plants under chilling stress [16, 38]. In this study, rootstocks promoted the expression of miR395a in grafted cucumber seedlings under chilling stress. miR395a was predicted to target *sulfate transport related proteins low affinity sulfate transporter 3* (*SUT3)*, *chloroplastic ATP sulfurylase 1* (*APS1)* and *sulfate transporter 2.1* (*SUT21)* to participate in sulfate transport in plants [37]. In addition, mir395a was predicted to target *gibberellin-regulatory protein 1* (*GASA1)*, which is involved in the plant response to abiotic stress by regulating gibberellin signaling [39].

miR397 can influence plant tolerance to chilling stress by regulating *CBF* expression [40]. In addition, miR397 overexpression enhances the growth of banana plants while maintaining their salt stress resistance [41]. In this study, ‘Yunnan figleaf gourd’-grafted cucumber seedlings up-regulated miR397a expression, which was predicted to target several chilling stress-related genes, including *LAC4*, *LAC11*, *LAC17* and *Thaumatin-like protein 1* (*TLP1)*. TLP1 is a high polyphenol oxidase (PPO) protein that can act as an antifreeze protein to improve chilling tolerance in plants [42–44]. Some studies have shown that miR398 is down-regulated by chilling stress, and it regulates intracellular ROS content by modulating CSD1 and CSD2 [45, 46]. In this study, rootstocks down-regulated the miR398a-3p expression, which was predicted to regulate ROS content by targeting *superoxide dismutase 9* (Cu-Zn) [47].

In addition, we predicted 6 and 8 novel miRNAs from TH/TC and TY/TC comparison pairs, respectively. Among these novel miRNAs, there were 5 identical novel miRNAs (novel-mir23, novel-mir26, novel-mir30, novel-mir37, and novel-mir46) in two comparison pairs. Novel-mir23 was predicted to target *deoxycytidine deaminase* (*DCTD)*, which can reduce the miscoding of intracellular genetic material by participating in the metabolism of intracellular cytosine nucleoside [48]. Novel-mir26 was predicted to target *late embryogenesis abundant protein D-34* (*LEA34)* and *cysteine-rich receptor-like protein kinase 10* (*CRK10)*, both of which play a role in intracellular ROS scavenging, protein protection and ABA signaling [49–51]. Novel-mir30 was predicted to target *cytoplasmic acetyl-CoA acetyltransferase 1* (*AACT1*) and *Telomere repeat-binding protein 5* (*TRP5*), with the function of *AACT1* remaining unclear and no significant difference in expression and metabolism between *AACT1* mutants [52]. TRP5 specifically binds to plant double-stranded telomere repeats (TTTAGGG)_n_ to reduce DNA damage and maintain normal cell development [53]. Novel-mir37 was predicted to target *gibberellin acid-insensitive 1* (*GAI1)*, a member of the DELLAs subfamily of the GRAS family and negatively regulates GA signaling. In addition, GAI1 can also bind to the ABF factor and is involved in plant ABA signal transduction in plants [54]. Novel-mir46 was up-regulated by two types of rootstocks and predicted to target *PME3*, which may be an important factor contributing to the promotion of cucumber seedling growth under chilling stress by rootstocks.

In response to chilling stress, novel-mir32 was up-regulated by ‘Huangchenggen No. 2’ and it was predicted to target *PME3* and *NAC*. NACs regulate plant chilling tolerance by participating in the ICE-CBF signaling pathway [55]. The expressions of novel-mir38, novel-mir65 and novel-mir78 were regulated by ‘Yunnan figleaf gourd’. Among them, novel-mir38 was down-regulated and was predicted to target cysteine-rich receptor-like protein kinase 29 (*CRK29)*. CRKs are involved in plant responses to various abiotic stresses by transmitting and sensing ROS/redox signals [56, 57]. Novel-mir65 was up-regulated by ‘Yunnan figleaf gourd’ and was predicted to target *SUT3*, *SUT21* and *APS1*, which are all involved in sulfate transport in plants [58]. Novel-mir78 was down-regulated by ‘Yunnan figleaf gourd’ and was predicted to target *putative pentatricopeptide repeat-containing protein* and *probable WRKY transcription factor 75* [59, 60].

### miRNAs associated with chilling tolerance affected by silicon nutrition in seedlings

We identified 6 significantly differentially expressed miRNAs in response to silicon from TC/TS comparison pairs. These including 2 known miRNAs (miR168a-5p and miR390a-5p) and 4 novel miRNAs (novel-mir26, novel-mir55, novel-mir67, novel-mir70). miR168 is induced by abiotic stresses such as drought and low temperature, which indirectly regulates the function of other miRNAs by targeting *argonaute1* (*AGO1*) [14, 61]. Up-regulation of miR168a has been reported to improve plant tolerance to drought stress, and the promoter region of miR168a contains ABA cis-acting elements that may be involved in the ABA signaling pathway [62]. In this study, silicon up-regulated miR168a-5p and was predicted to target *AGO1*. AGO1 associates with miRNAs to form an RNA-induced silencing complex (RISC), which is involved in target RNA cleavage and mediates post-transcriptional gene silencing [60].

miR390 plays a crucial role in regulating plant growth and abiotic stress response through the auxin signaling pathway by regulating the auxin response factor (ARF) [63, 64]. In this study, we observed that silicon increased the expression of miR390, which was predicted to target *leucine-rich repeat receptor-like tyrosine-protein kinase PXC3* and *E3 ubiquitin-protein ligase MARCH3*. PXC3 is a member of the receptor-like protein kinase (RLK) subfamily, which binds to extracellular signaling molecules and activates intracellular kinase domains, thereby completing transmembrane signal transduction [65]. RLKs have been shown to be involved in plant responses to abiotic stresses, such as low temperature and drought, by regulating mitogen-activated protein kinase (MAPK) cascade, ABA and ROS signaling [66, 67]. MARCH3 is a crucial component of the ubiquitin-26S proteasome system (UPS) and plays an essential role in plant response to abiotic stress [68, 69]. For example, E3 ubiquitin-protein ligase is up-regulated by low temperature, which attenuates the damage caused by chilling stress in rice [70].

In addition, 4 novel miRNAs (novel-mir26, novel-mir55, novel-mir67 and novel-mir70) were predicted to respond to exogenous silicon. Notably, novel-mir26 was responsive to both silicon and two rootstocks and was predicted to target *late embryogenesis abundant protein 34* (*LEA34*) and *cysteine-rich receptor-like protein kinase 10* (*CRK10*). LEA34 and CRK10 may be involved in intracellular ROS scavenging, protein protection and ABA signaling [71]. Under chilling stress, silicon reduced the expression of novel-mir55 and novel-mir70, which were predicted to target *expansin A23* (*EXPA23)*. EXPA23 is a member of the expansin superfamily, which disrupts non-covalent bonds between cell wall polysaccharides and leads to stress-dependent cell expansion in response to various abiotic stresses [72, 73]. Overexpression of *EXPA8-B* and *EXPA8-D* has been shown to improve the chilling tolerance in Arabidopsis [74]. Under chilling stress, novel-mir70 was up-regulated by exogenous silicon and was predicted to target the transcription factor *GTE12*. Although twelve GTE proteins have been found in Arabidopsis thaliana, their functions are not well understood.

## Conclusion

In this study, we used high-throughput sequencing technology to identify miRNAs that respond to low temperature, exogenous silicon and two rootstocks in cucumber seedlings. Our results showed that novel-mir46 and miR398a-3p were observed in TC/CC, TH/TC and TY/TC comparison pairs, indicating that these miRNAs respond not only to chilling stress but also to the rootstocks. However, we did not identify the same miRNAs in TC/CC and TS/TC comparison pairs. Furthermore, the differential expression of novel-mir38, novel-mir65, novel-mir78 and miR397a could be an important factor contributing to the differences in chilling tolerance of grafted cucumbers caused by two rootstocks. The identification of these miRNAs helps us to understand the differences in molecular mechanisms of chilling stress mitigation between rootstocks with different de-blooming capacities, which is essential for the subsequent research to improve the chilling tolerance of grafted cucumber.

## Methods

### Plant materials and treatments

The scion was ‘Xintaimici’ cucumber (*Cucumis sativus* L.), and the rootstocks were ‘Huangchenggen No. 2’ (*Cucurbita moschata* D.) with strong de-blooming capacity and ‘Yunnan figleaf gourd’ (*Cucurbita ficifolia* B.) with weak de-blooming capacity. The experiment was conducted from June 2021 to April 2022 in an artificial climate chamber at Shandong Agricultural University. Cucumber seeds and rootstock seeds were immersed in water at 30°C for 4 h and 6 h, respectively, and placed in an incubator (HZQ-F160a, Yiheng, China) to stimulate germination for 24 h and 48 h at 28 ± 1°C under humid (relative humidity between 85% and 100%) and dark conditions, respectively. The germinated seeds were sown into available seedling substrate plugs (peat: vermiculite: perlite = 1:1:1 (v:v:v)). When the rootstock seeds germinated, the scion seeds were sown into the plugs. When the cotyledon of the scion and the first true leaf of the stock had fully expanded, the seedlings were grafted by using ‘hole insertion’ method. To increase the survival rate of the grafted cucumber seedlings, the temperature was maintained at 28°C /24°C (day/night) and the relative humidity was kept between 85% and 100% for about 7 days. When the grafted seedlings survived, they were transferred to an environment with a temperature of 28°C/18°C, relative humidity of 75%~85% and light intensity of 400 µmol·m^− 2^·s^− 1^ for further cultivation. When the first true leaves of self-rooted cucumber seedlings and grafted cucumber seedlings spread out, 18 healthy seedlings with similar growth were selected and placed in 3 incubators, each containing 6 L of Yamazaki cucumber nutrient solution. The solution was replaced every 3 days.

The experiment consisted of five treatments: (1) CC: Self-rooted cucumber seedlings were grown in nutrient solution and maintained at 28 ± 1 °C/18 ± 1 °C. (2) TC: Self-rooted cucumber seedlings were grown in nutrient solution and maintained at 10 ± 1 °C/5 ± 1 °C. (3) TS: Self-rooted cucumber seedlings were grown in nutrient solution containing 0.5mmol·L^− 1^ Na_2_SiO_3_·9H_2_O and maintained at 10 ± 1 °C/5 ± 1 °C. (4) TH: ‘Huangchenggen No. 2’-grafted cucumber seedlings were grown in nutrient solution and maintained at 10 ± 1 °C/5 ± 1 °C. (5) TY: ‘Yunnan figleaf gourd’-grafted cucumber seedlings were cultured in nutrient solution and maintained at 10 ± 1 °C/5 ± 1 °C. TC served as the controlled experiment among the five treatments. After 12 and 24 h of treatment, respectively, 8 seedlings were randomly selected for sampling. The sampling methods were as follows: the third leaf was taken, the veins were removed, mixed well, and cut into pieces. Samples were frozen in liquid nitrogen and immediately stored at -80 °C.

### Determination of growth index

The plants were exposed to the low temperature of 10 ± 1 °C/5 ± 1 °C (day/night) for 2 days, and 8 plants were randomly selected from each treatment group to measure the growth index of cucumber seedlings. The length of cucumber cotyledon to growing point was measured with a ruler as plant height, and the diameter of cucumber cotyledon node position was measured with a vernier caliper as stem diameter. After measuring the plant height and stem diameter, the seedlings were divided into roots and shoots with scissors, washed and blotted with deionized water, and the fresh weight of the branches was determined. The third functional leaf above the cotyledon of cucumber seedlings was taken. The leaf area was measured using LA-S root scanner (WSeen, China) and WinRHIZO image analysis software. The shoots were then placed in an oven and kept at 105 °C for 30 min, and then the temperature was lowered to 75 °C until they maintained a constant weight, and the dry weight of shoots was recorded.

### Determination of chilling injury index

After 24 h of chilling stress, the chilling injury index of cucumber seedlings was evaluated according to the chilling injury index classification established in a previous report [75]. The chilling injury index was calculated as follows: Grade 0: No injury symptoms. Grade 1: The edge of the first leaf is yellow or slightly dehydrated. Grade 2: Dehydration spots appear on a small portion of the first leaf; other leaves are slightly dehydrated. Grade 3: Dehydration spots appear on the first half of the leaves; other leaves are slightly dehydrated. Grade 4: Desiccated spots appear on most of the leaf area; the other half of the leaves are dehydrated. Grade 5: Almost all leaves are severely dehydrated and wilted. Chilling injury index = Σ (plants of different grades × grade) / (total plants × maximum grade).

### Small RNA libraries construction and sequencing

Total RNA was extracted from cucumber leaves of 8 plants in 3 replicates per treatment using Trizol reagent (Vazyme, Nanjing, China). The degree of RNA degradation and contamination was analyzed by agarose gel electrophoresis, and then the purity of RNA samples was detected by NanoDrop (D260 nm/D280 nm ≥ 1.8 × OD260, OD230 ≥ 1.0). The Qubit detection method was used to accurately quantify the concentration of RNA samples (total RNA concentration ≥ 250 ng/µL); Agilent 2100 was used to accurately detect the integrity of RNA. Finally, the sRNA library was produced by using samples with RNA integrity ≥ 8. Agilent 2100 and other methods were used to accurately detect the integrity of RNA. Finally, high-throughput sequencing was performed on the HiSeq2000 instrument (Illumina, USA), and each treatment was sequenced three times.

### Raw data pre-processing

The raw sequencing data contained low-quality reads, which were filtered for subsequent analysis [76]. Data processing methods are as follows: (1) Remove the low-quality reads (the quality value sQ ≤ 20 accounts for more than 30% of the total reads). (2) Remove the reads with a proportion of N was greater than 10. ‘N’ indicates that the base cannot be determined. (3) Remove the reads with 5` primer contamination. (4) Remove the reads with poly A/T/G/C. (5) Remove the reads shorter than 18 nt. The clean reads with 18 nt to 30 nt were obtained, and the unique reads and total reads were calculated. Finally, the length distribution of clean reads in each library is calculated.

### Identification of known miRNAs and novel miRNAs in cucumber leaves

The 18 nt to 30 nt length sRNAs were mapped to Cucumber (Chinese Long) v3 genome [77] by Bowtie (no mismatch), then their expression and distribution on reference sequence were analyzed. To remove tags from protein-coding genes, repetitive sequences, rRNA, tRNA, snRNA and snoRNA, small RNA tags were mapped to Repeat Masker (http://repeatmasker.org/cgi-bin/WEBRepeatMasker), Rfam database (http://rfam.janelia.org/) and GenBank database (http://ftp.ncbi.nlm.nih.gov/genbank/). The remaining unique sequences were matched to miRbase 22.1 database (https://www.mirbase.org/sea-rch.shtml) using BLAST to identify conservative and known miRNAs in cucumber (no mismatch). Perfect match between sRNA and precursor sequences. However, reads with at least 16 nt overlap consistent with mature miRNAs in miRBase were allowed to offset. Count the number of miRNAs matching the conditions and analyze the base deviations at each position of all identified miRNAs.

To predict novel miRNAs, we utilized miREvo software to align reads with the reference genome and predict miRNA precursors [78]. Briefly, we performed a 70 nt sliding window based on the positions of reads mapped to reference genome to determine the suitable location, from which we extracted the upstream 20 nt and downstream 70 nt to predict the secondary structure of the miRNA precursor. Next, we analyzed the predicted precursor sequences using the miRDeep2 software, which was accessed through the miREvo interface [79]. After obtaining the precursor sequences, we applied a series of selection criteria to filter out unsuitable candidates, including (1) no branched secondary structures upon folding; (2) categorization of precursor sequences into mature, loop, or star strands based on mapping results; (3) at least 60% of the mature strand sequence being covered by reads. Using the predicted mature regions of the precursor sequences, we identified novel sRNA sequences. We used miREvo_v1.1 with the following primary parameters: -i, -r, -M, -m, -k, -p 10, -g 50,000; and miRDeep2_0_0_5 with the following primary parameters: quantifier.pl, -p, -m, -r, -y, -g 0, -T 10.

### Analysis of differentially expressed miRNA

The expression of miRNAs in the sample was counted, and the expression level was normalized using the TPM algorithm (actual miRNA count / total count of clean reads × 1,000,000). Then, we used the DESeq2 software package (3.0.3) to analyze the differential expression between two groups. The *p*-value ≤ 0.05 and | log2 (foldchange) | ≥ 1 were set as the threshold for screening differentially expressed genes, and the *p-*value was calculated according to Benjamini & Hochberg method. The results of four comparison pairs were obtained by difference analysis. The differentially expressed miRNAs in TC/CC comparison pair might be related to low-temperature response, and the differentially expressed miRNAs in TS/TC comparison pair might be related to the response to exogenous silicon under chilling stress. The differentially expressed miRNAs in TH/TC comparison pair might be related to the induction of the ‘Huangchenggen No. 2’ under chilling stress. The differentially expressed miRNAs in TY/TC comparison pair might be related to the induction of ‘Yunnan figleaf gourd’ under chilling stress.

### Prediction and analysis of miRNAs target genes

The miRNA sequence was compared with the genomic EST sequence of cucumber, and TargetFinder predicted the target genes. To further understand the biological function of the target genes, we mapped all candidate genes to GO terms in the database (http://www.geneontology.org/) and performed quantitative statistics. Compared with the internal reference gene background, a hypergeometric test was used to find the significantly enriched GO terms in candidate target genes. The GO term with *p-value* ≤ 0.05 is defined as significantly enriched in candidate target genes. Similarly, all candidate genes were mapped to KEGG [80, 81] pathways in database and determined the significantly enriched KEGG metabolic pathways in target gene candidates were determined by hypergeometric test (*p*-value ≤ 0.05).

### Identification of miRNAs and target gene expression by qRT-PCR

Eight plants were randomly selected from each treatment group, and the materials were mixed and subjected to qRT-PCR analysis three times for each treatment group. The differentially expressed miRNAs were verified by qRT-PCR using the miRNA 1st Strand cDNA Synthesis Kit (by STEM-Loop) detection Kit (Vazyme, Nanjing, China). The volume of each reaction was 20 µL, including 10 µL of 2 × miRNA Universal SYBR qPCR Master Mix, 0.4 µL of Specific Primer (10 µM), 0.4 µL of universal reverse primer (10 µM) and 2 µL of Template cDNA. The PCR program was as follows: The pre-denaturation was performed at 95 °C for 5 min, cycling was performed at 95 °C for 40 cycles (5 s per cycle), and the melting curve was generated at 60 °C for 30 s. Finally, the data were calculated using the 2^−ΔΔCt^ method (U6 snRNA as the reference gene).

The expression level of mRNA was verified by qRT-PCR. The HiScript II One Step RT-PCR kit (Vazyme Biotech, Nanjing, China) was used to prepare the first strand cDNA according to the instructions. qRT-PCR was performed on the ABI 75,000 Real-Time PCR machine (Applied Biosystems, Foster City, CA, USA). The reaction volume of qPCR was 20 µL, including 10µL of SYBR Premix EX TaqII, 0.4 µL of forward primers (10 µM), 0.4 µL of reverse primers (10 µM), and Template cDNA of 2 uL. The PCR programme was as follows: The predenaturation was performed at 95 °C for 30 s, cycling was performed at 95 °C for 40 cycles (5 s per cycle), and the melting curve was generated at 60 °C for 30 s. Finally, the 2^−ΔΔCt^ method was used to calculate the data (actin as the reference gene). Additional file 5: Table S5 lists the sequences of the primers.

## Electronic supplementary material

Below is the link to the electronic supplementary material.

Supplementary Material 1

Supplementary Material 2

Supplementary Material 3

Supplementary Material 4

Supplementary Material 5

## Data Availability

The datasets generated and analyzed during the current study are available in the NCBI Sequence Read Archive (SRA) repository, at. https://www.ncbi.nlm.nih.gov/bioproject/938179 and accession #: PRJNA938179.
